# Altered Blood Biomarker Profiles in Athletes with a History of Repetitive Head Impacts

**DOI:** 10.1371/journal.pone.0159929

**Published:** 2016-07-26

**Authors:** Alex P. Di Battista, Shawn G. Rhind, Doug Richards, Nathan Churchill, Andrew J. Baker, Michael G. Hutchison

**Affiliations:** 1 Institute of Medical Science, University of Toronto, Toronto ON, Canada; 2 Defence Research and Development Canada, Toronto Research Centre, Toronto, ON, Canada; 3 Faculty of Kinesiology & Physical Education, University of Toronto, Toronto ON, Canada; 4 Keenan Research Centre for Biomedical Science of St. Michael’s Hospital, Toronto, ON, Canada; 5 Departments of Critical Care, Anesthesia and Surgery, St. Michael’s Hospital, University of Toronto, Toronto ON, Canada; 6 Keenan Research Centre, Li Ka Shing Knowledge Institute, St. Michael’s Hospital, Toronto, ON, Canada; University of Victoria, CANADA

## Abstract

The long-term health effects of concussion and sub-concussive impacts in sport are unknown. Growing evidence suggests both inflammation and neurodegeneration are pivotal to secondary injury processes and the etiology of neurodegenerative diseases. In the present study we characterized circulating brain injury and inflammatory mediators in healthy male and female athletes according to concussion history and collision sport participation. Eighty-seven university level athletes (male, n = 60; female, n = 27) were recruited before the start of the competitive season. Athletes were healthy at the time of the study (no medications, illness, concussion or musculoskeletal injuries). Dependent variables included 29 inflammatory and 10 neurological injury analytes assessed in the peripheral blood by immunoassay. Biomarkers were statistically evaluated using partial least squares multivariate analysis to identify possible relationships to self-reported previous concussion history, number of previous concussions and collision sport participation in male and female athletes. Multiple concussions were associated with increases in peripheral MCP-1 in females, and MCP-4 in males. Collision sport participation was associated with increases in tau levels in males. These results are consistent with previous experimental and clinical findings that suggest ongoing inflammatory and cerebral injury processes after repetitive mild head trauma. However, further validation is needed to correlate systemic biomarkers to repetitive brain impacts, as opposed to the extracranial effects common to an athletic population such as exercise and muscle damage.

## Introduction

Concern regarding the potential negative health impact of concussions and collision sport participation has led to an increased demand to delineate the pathophysiological mechanisms mediating long-term outcomes [1]. Our current conceptual understanding of concussion pathophysiology consists of an acute disturbance of neurobehavioral function together with damage to neuronal and glial cells [2]. Symptoms are commonly short-lived and self-limited, resolving within a span of days to weeks [3–5]; however, recent objective advances in neuroimaging and analytical biomarker assessment have documented underlying functional and structural abnormalities persisting beyond symptom resolution [6–8]. Furthermore, evidence is now emerging that suggests concussion, as well as the repetitive head impacts that commonly occur in collision sport participation, may contribute to negative health outcomes such as chronic traumatic encephalopathy (CTE) [9–14]. However, our current understanding of these pathophysiological processes in humans is limited.

Inflammation is an important contributor to both repair and neurodegenerative processes after neurotrauma [15–17]. Resident microglial cells and central nervous system (CNS) invading peripheral immune cells facilitate the acute repair and regeneration of damaged brain tissue via the release of neurotrophic factors and scavenging of debris [18–20]. However, chronic inflammation may also exacerbate neuronal and glial cell injury, leading to further cellular degeneration and culminating in the deposition of neurofibrillary tangles and amyloid plaques [18]. In view of this, human studies have found prolonged neuroinflammation persisting for months to years after moderate and severe traumatic brain injury (TBI) [21–24], and experimental evidence suggests these maladaptive processes may occur through the interaction of inflammatory mediators and glutamate receptors in the CNS [25–28]. Moreover, multiple head impacts may worsen these processes by priming microglial cells, leading to an exaggerated inflammatory reaction upon subsequent trauma [18,29].

Inflammation post-concussion is difficult to characterize due to practical limitations such as the inability to access tissue proximal to the site of injury, and the invasive nature of cerebral spinal fluid (CSF) acquisition [30]. Nevertheless, peripheral blood samples have the potential to provide meaningful information regarding inflammatory processes both in the CNS and periphery in response to brain injury, in a relatively cost-effective, non-invasive manner [18,30,31]. In view of this, recent evidence has shown that increased circulating C-reactive protein levels post-injury are associated with persistent post-concussive syndrome symptoms [32], and coated platelet levels, an inflammatory correlate, are elevated in mild TBI patients up to 9 years post-injury [33].

Historically, one of the limitations in concussion research has been the lack of consideration for potential sex differences. Specifically, there has been a paucity of concussion research on females [34]. Yet, available evidence suggests that females may be at a greater risk for concussion [35,36], report more symptoms post-concussion [35,37], and take longer to recover [35,38]. In addition, it is known that males and females display distinct immunological responses; women exhibit stronger cellular and humoral immune responses, are more prone to many autoimmune diseases, but are less susceptible to various of bacterial, viral, and fungal infections [39,40]. Therefore, the possibility exists that inflammatory related processes occurring chronically after concussion may have sex-specific pathological sequelae.

Thus, in this study we set out to examine a panel of systemic brain injury markers and inflammatory mediators in a sample of male and female athletes to characterize the relationship between these biological indices, concussion history, and collision sport participation.

## Methods

### Participants

Participants were recruited from University of Toronto intercollegiate “varsity” athletic teams between August 2014 and December 2015. A member of the research team provided an overview of the study and requested consent to obtain blood samples and use the Sport Concussion Assessment Tool 3 (SCAT3) results for research purposes. Medical history was obtained by the team’s therapist/trainer, followed by administration of the SCAT3. Sixteen teams (8 male, 8 female) were contacted for research purposes, including the following sports: basketball, baseball, field hockey, football, ice hockey, lacrosse, rugby, soccer, wrestling and volleyball. Athletes were excluded if they suffered from seasonal allergies, cold, infection, disclosed any inflammatory-related health conditions, were taking any medications other than birth control at the time of the study, or had musculoskletal injuries (9 subjects). Study procedures were approved by the Health Sciences Research Ethics Board, University of Toronto (protocol reference # 27958), and all participants provided written informed consent prior to the study.

### Measures

*Sport Concussion Assessment Tool 3 (SCAT3)*: The SCAT3 combines aspects of several previously published concussion tools into eight components designed to assess concussion symptoms (number endorsed and severity), cognition (Sideline Assessment of Concussion or SAC and Maddocks questions), balance (firm conditions of the Balance Error Scoring System or BESS), Glasgow Coma Scale (GSC) and neurological signs (physical signs, coordination) [41]. Each of the eight components are scored and recorded. The symptom score is comprised of a 22-item post-concussion symptom scale using a seven-point Likert scale rating. Symptom severity is obtained by summing the rated symptom score for each symptom [39]. This symptom scale has been shown to be reliable and valid for the assessment of both symptom presence and severity [37,41,42].

### Blood Sample Collection

Venous blood samples were drawn from athletes after consent was obtained and prior to the beginning of the competitive varsity season. Samples were drawn into a 10-mL K_2_EDTA (with 4mM sodium metabisulfite [Na_2_S_2_O_5_]) or 4-mL non-additive (Vacutainer, Becton Dickinson, NJ, USA) tube. Within one hour, specimens were centrifuged at 1600 x *g* for 15 minutes at 4°C, and the plasma supernatant was aliquoted and frozen at -70°C until analysis.

### Biomarker Analysis

Twenty-eight of the thirty-nine markers were analyzed using MSD^®^ 96-Well MULTI-ARRAY/-SPOT^®^ V-plex Human Immunoassay Kits purchased from MSD (MD, USA), and run on a Meso-Scale Discovery (MSD^®^) Sector imager^TM^ 6000 with Discovery Workbench software (version 3.0.18). A prototype assay panel of eleven additional neuroinjury markers including total tau, glial fibrillary acidic protein (GFAP), s100 calcium-binding protein (s100) B, neuron specific enolase (NSE), Neurogranin (NRGN), creatine kinase-BB isoenzyme (CKBB), visinin-like protein (VILIP)-1, von Willebrand factor (vWF), brain derived neurotrophic factor (BDNF), peroxiredoxin (PRDX)-6, and monocyte chemoattractant protein (MCP)-1, was assessed by multiplexed immunoassay [43].

### Statistical Analyses

Demographic and descriptive statistics were completed on male and female athletes by student’s independent *t-*test Mann Whitney *U*, or χ^2^, where appropriate. For dichotomized analysis of collision vs. non collision sports, collision sports were delineated as sports with purposeful contact as an inherent part of the game, and included men’s ice hockey, football, rugby, lacrosse, and women’s rugby [44]. All other sports, including those where inadvertent contact may occur (soccer, basketball), were considered non-collision sports [44]. For all analyses, individual biomarker values were excluded if they were above or below the manufacturers’ recommended level of quantitation for each analyte, or displayed a coefficient of variance >25% between duplicates. Because multiple 96-well plates were analyzed, inter-plate variance was accounted for; plates were only included in the statistical analysis if the inter-plate variance was <20%, calculated from internal control samples acquired on each plate. Biomarkers were not included in the multivariate analysis if >30% of the data points were missing in any group. Multivariate analysis was conducted using a partial least squares discriminant (PLS-DA). PLS-DA is a supervised technique used to objectively characterize the covariance between a set of predictor variables and binary response variables [45,46]. A PLS-DA output provides model prediction accuracy (Accur) and posterior probability (PProb). Briefly, these indices measure how accurately a fitted model can predict a binary outcome based solely on predictor variables. Accur is evaluated by assigning each subject to the outcome group with the most similar mean PLS score; 1 = correctly predicted, and 0 = incorrectly predicted. This provides a simple, robust metric of prediction, which does not depend on a specific probability model. PProb is the likelihood of the PLS model identifying the correct outcome conditional on the observed subject scores, under a Gaussian noise model. This provides an alternative probabilistic measure that accounts for uncertainty in the PLS model and observed data. With numerous response variables, the PLS analysis yields the fraction of variance explained. Fraction of variance reflects the proportion of total inter-subject variability in biomarker data that is described by the PLS component of interest. In the current study, covariance between peripheral blood biomarkers (predictor variables) and both concussion history and collision sport participation (response variables) was assessed separately in male and female athletes. Missing biomarker values were imputed using the k-means nearest-neighbour method [47], and were rank-transformed to ensure robustness against non-normality. Significant biomarker loadings were identified by performing bootstrap resampling on subjects (1000 iterations) to obtain empirical p-values, which were then corrected for multiple comparisons at a false discovery rate (FDR) of 0.05. For PLS plots, variable loadings are represented as bootstrap ratios (i.e., the bootstrapped mean / standard error), which are z-scored statistics reflecting the reliability of variable contributions. Descriptive and univariate statistics were completed using Stata Version 14.1 (StataCorp, TX, USA). Multivariate analyses were conducted using in-house software developed for Matlab, Version R2015b (Matworks, Natick MA). All data were visualized using GraphPad Prism Version 6.0f (GraphPad Inc., CA, USA).

## Results

### Demographics and Clinical Characteristics

A total of 87 athletes were included in the study (male, n = 60; female, n = 27). Athlete characteristics and concussion history are listed in Table 1. Briefly, athletes were of similar age, and we observed no significant differences in medical history and SCAT3 symptoms at the time of the study. There were no differences between male and female athletes regarding concussion history, number of previous concussions, and days since last concussion. As expected, a significantly higher proportion of males played in collision sports as compared to their female counterparts (63.9% vs. 7.4%, respectively).

**Table 1:** Athlete demographics and characteristics.

Characteristic	Male (n = 60)	Female (n = 27)	P value
Age (years)	19.5 ± 2.0	19.5 ± 1.8	0.86
Concussion history–n (%)	23 (38.3)	12 (44.4)	0.55
Days since last concussion–median (IQR)	793 (420–1249)	552 (375.5–714.5)	0.170
Number of previous concussions	0.64 ± 1.0	1.1 ± 1.7	0.619
0 –n (%)	37 (61.7)	15 (55.6)	
1 –n (%)	12 (20.0)	5 (18.5)	
2 –n (%)	8 (13.3)	3 (11.1)	
≥ 3 –n (%)	3 (5.0)	4 (14.8)	
Collision sport participation–n (%)	39 (65.0)	2 (7.4)	**<0.001**
Medical history–n (%)			
Migraines	2 (3.3)	0 (0.0)	0.156
Learning disability	1 (1.7)	0 (0.0)	0.203
Depression/Anxiety or other psychiatric disorders	1 (1.7)	2 (7.4)	0.226
Family history of psychiatric illness	12 (20.0)	7 (25.9)	0.247
SCAT3 symptom scores			
Total symptoms	3.4 ± 3.6	3.8 ± 3.0	0.350
Symptom severity	5.4 ± 7.0	6.0 ± 4.7	0.15

Unless otherwise stated, results are reported as the mean ± standard deviation (SD).

Demographic and characteristic differences between male and female athletes were assessed by χ^2^, Mann-Whitney *U*, or independent student’s *t*-test, where appropriate.

### Systemic inflammatory marker analysis

A list of all biomarkers with corresponding median values and the percent of samples detectable in the plasma for each analyte are listed in Table 2. No significant differences were identified between male and female athletes who did not participate in collision sports or who had no previous history of concussion (data not shown).

**Table 2:** List of biomarkers analyzed.

Markers (pg/mL)*	% Quantifiable^a^	Median (IQR)
*Cytokines*		
IL-1α	27.6	—
IL-1β	0	—
IL-2	0	—
IL-4	1.1	—
IL-5	0	
IL-6	2.3	—
IL-7	59.8	2.6 (2.0–3.7)
IL-10	6.9	—
IL-12p40	97.7	121.4 (93.1–146.2)
IL-12p70	0	—
IL-13	0	—
IL-15	100	2.3 (2.0–2.7)
IL-16	70.1	259.2 (198.5–369.0)
IL-17A	1.1	—
TNF-α	96.5	1.8 (1.5–2.2)
TNF-β	0	—
GM-CSF	0	—
VEGF	87.3	36.5 (28.0–55.6)
IFN-γ	16.1	—
*Chemokines*		
Eotaxin	91.9	77.7 (62.7–94.3)
Eotaxin-3	69.3	22.2 (18.5–31.3)
IP-10	77.0	202.6 (159.7–257.1)
IL-8	82.8	1.9 (1.5–2.7)
MCP-1	96.6	86.8 (72.4–109.4)
MCP-4	94.2	26.5 (19.5–38.3)
MDC	98.8	807.2 (706.3–989.1)
MIP-1α	4.6	—
MIP-1β	95.4	37.9 (30.3–49.6)
TARC	88.5	43.1 (27.3–55.5)
*Neuroinjury Markers*		
s100B	85.0	707.1 (603.0–896.1)
GFAP	59.8	75.6 (63.2–98.1)
NSE (ng/mL)	100	1.5 (1.2–2.1)
Neurogranin (ng/mL)	100	7.8 (4.5–11.8)
CKBB	11.5	—
VILIP-1	8.0	—
Tau	98.8	24.0 (18.5–32.9)
vWF (μg/mL)	96.5	38.2 (23.3–53.5)
BDNF	100	856.4 (566.4–2022.7)
PRDX-6 (ng/mL)	100	26.4 (18.6–33.2)

Interleukin (IL)-1α, -1β, -2, -4, -5, -6, -7, -10, -12p40, -12p70, -13, -15, -16, -17A, tumor necrosis factor (TNF) -α, -β, granulocyte macrophage colony-stimulating factor (GM-CSF), vascular endothelial growth factor (VEGF), interferon-gamma (IFN-γ), eotaxin, eotaxin-3, interferon gamma-induced protein (IP) -10, IL-8. monocyte chemoattractant protein (MCP)-1, -4, macrophage derived chemokine, (MDC), macrophage inflammatory protein (MIP)-1α, -1β, thymocyte- and activation-regulated chemokine (TARC), s100 calcium binding protein beta (s100B), glial fibrillary acidic protein (GFAP), neuron specific enolase (NSE), creatine kinase-BB isoenzyme (CKBB), visinin-like protein (VILIP-1), von Willebran factor (vWF), brain derived neurotrophic factor (BDNF), peroxiredoxin (PRDX) -6.

* = all markers reported as pg/mL unless otherwise stated

^a^ = Biomarkers were included if replicates had less than a 25% CV, were within the LLOQ and ULOQ, and had an inter-plate variance of less than 20% as measured by internal controls.

“—” = below assay quantitation in ≥50% of samples analyzed.

#### Multivariate analysis

**Fig 1:**
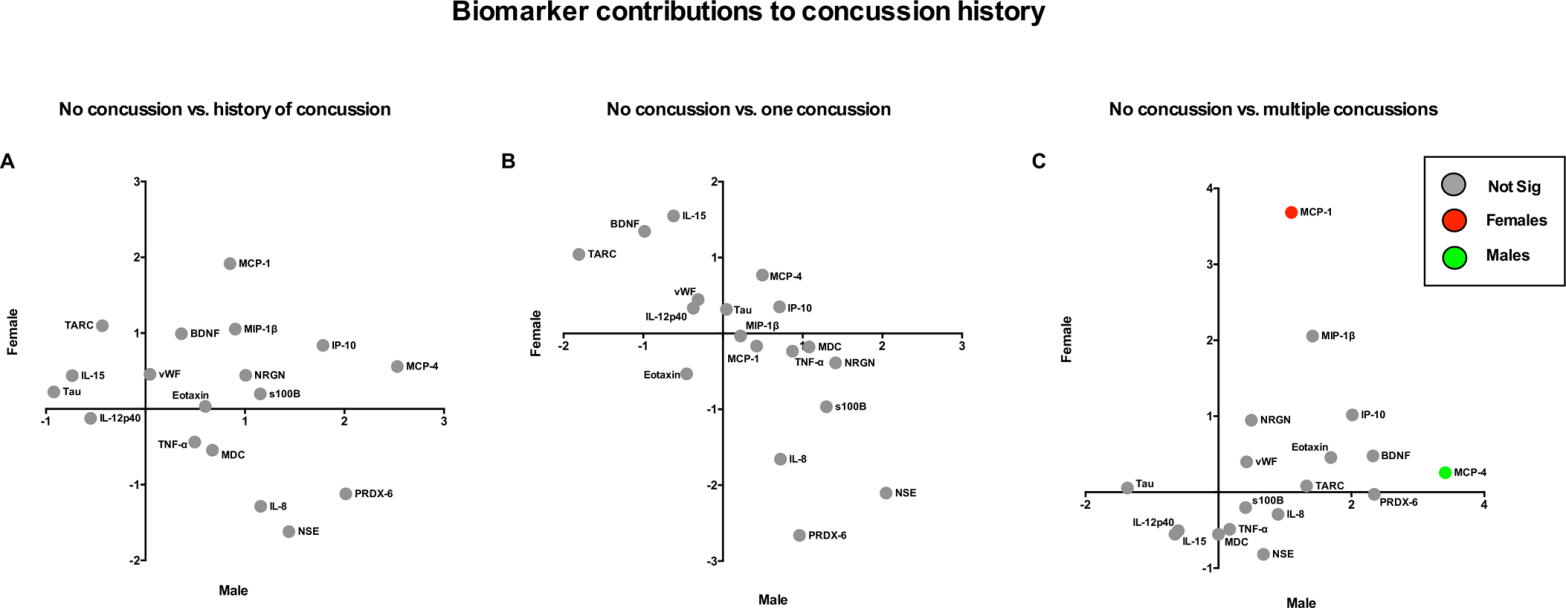
Biomarker covariance with concussion history in athletes. *brain injury markers*: s100 calcium-binding protein B (s100B), neuron specific enolase (NSE), Neurogranin (NRGN), tau, von Willebrond factor (vWF), brain derived neurotrophic factor (BDNF), peroxiredoxin (PRDX)– 6; *inflammatory markers*: interleukin (IL) -12p40, -15, tumor necrosis factor (TNF) -α, IL-8, monocyte chemoattractant protein (MCP)-1, -4, interferon gamma induced protein (IP) -10, macrophage derived chemokine (MDC), macrophage inflammatory protein (MIP)-1β, thymus and activation regulated chemokine (TARC), eotaxin. Blood biomarker contributions are displayed on the x-axis for males, and y-axis for females, in (A) healthy athletes with vs. without a history of concussion, (B) healthy athletes with a single previous concussion vs. no history of concussion, and (C) healthy athletes with multiple previous concussions vs. no history of concussion. Dots represent z-scores derived from individual bootstrapped loadings divided by the standard error of the mean. FDR = 0.05.

PLS analysis of the covariance between peripheral blood biomarkers and athlete characteristics is shown in Fig 1. No individual biomarkers were significantly correlated to previous concussion history in either male (model PProb = 0.50, Accur = 0.48) or female (model PProb = 0.41, Accur = 0.35) athletes (Fig 1A). Similarly, when further stratified, compared to athletes with no concussion history, athletes with one previous concussion displayed no significant differences in biomarker levels (males–model PProb = 0.47, Accur = 0.46; females–model PProb = 0.46, Accur = 0.46) (Fig 1B). However, in athletes with multiple previous concussions vs. those with no previous concussions (males–model PProb = 0.53, Accur = 0.51; females–model PProb = 0.43, Accur = 0.44), female athletes had significantly higher MCP-1 (median conc.; 96.4 vs. 69.3 pg/mL) levels, while male athletes had significantly higher MCP-4 (median conc.; 48.3 vs. 26.1 pg/mL) (Fig 1C). See S1 Table. for plasma concentrations of all biomarkers according to concussion history.

**Fig 2:**
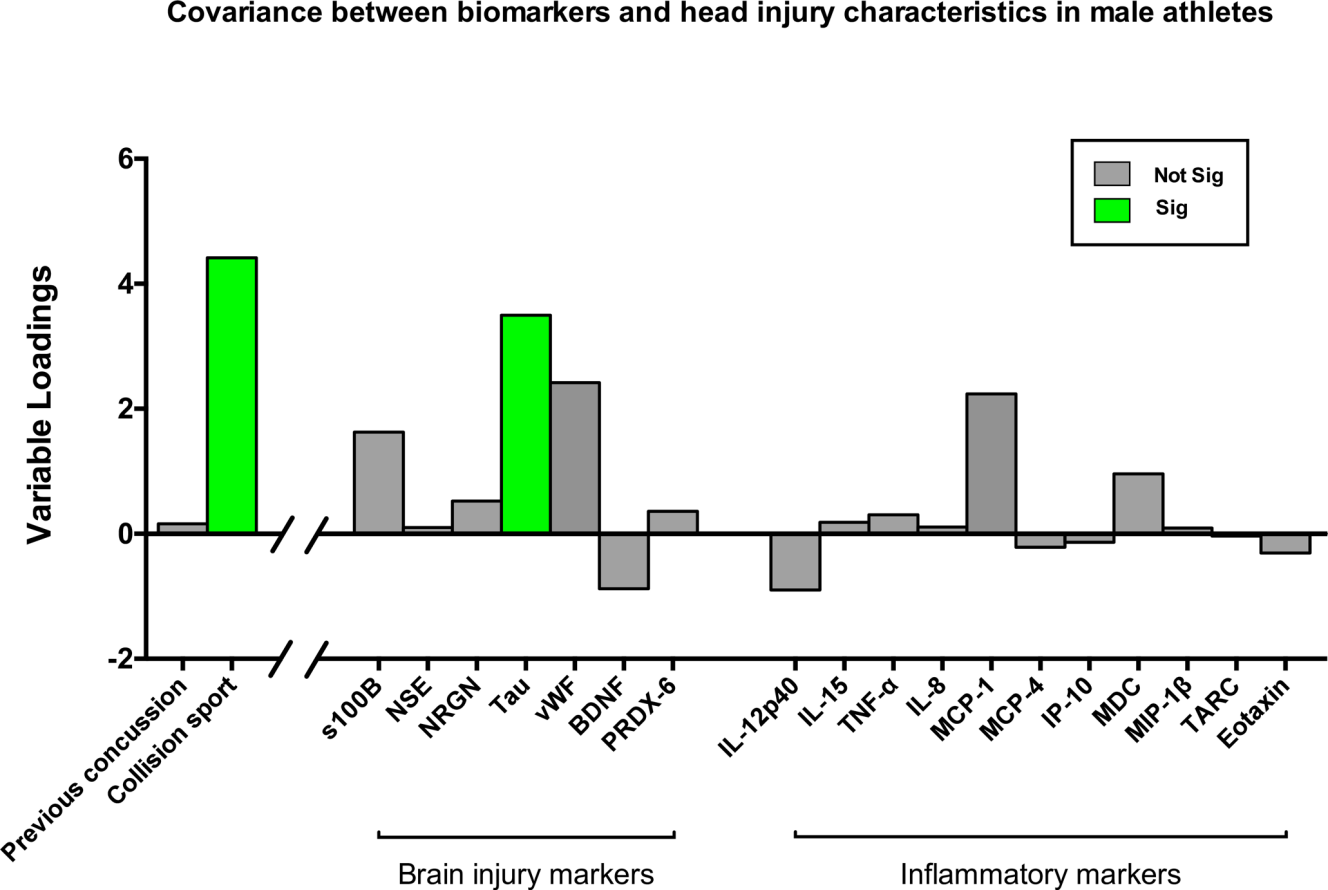
Covariance between biomarkers and head injury characteristics in male athletes. *brain injury markers*: s100 calcium-binding protein B (s100B), neuron specific enolase (NSE), Neurogranin (NRGN), tau, von Willebrond factor (vWF), brain derived neurotrophic factor (BDNF), peroxiredoxin (PRDX)– 6; *inflammatory markers*: interleukin (IL) -12p40, -15, tumor necrosis factor (TNF) -α, IL-8, monocyte chemoattractant protein (MCP)-1, -4, interferon gamma induced protein (IP) -10, macrophage derived chemokine (MDC), macrophage inflammatory protein (MIP)-1β, thymus and activation regulated chemokine (TARC), eotaxin. Bars represent z-scores derived from individual bootstrapped loadings divided by the standard error of the mean. FDR = 0.05.

PLS analysis of the covariance between systemic biomarkers, and both collision sport participation and previous concussion history in males is shown in Fig 2. Only collision sport participation significantly co-varied with increases in tau (median conc; 33.9 vs. 20.8 pg/mL in non-collision sport athletes). See S2 Table. for plasma concentrations of all biomarkers according to collision sport participation.

## Discussion

In this study we identified differences in the systemic biomarker profiles of male and female athletes who sustained multiple previous concussions, and in males who participate in collision sports. We included blood samples from athletes with no inflammatory-related conditions, musculoskeletal injuries or concussion symptoms prior to the start of the competitive season. To our knowledge, this is the first report to combine an array of brain injury-related and inflammatory indices chronically after sport concussion in male and female athletes.

We found healthy female athletes with a reported history of multiple concussions had elevated blood MCP-1 levels, while males had elevations in MCP-4. Chemokines are important facilitators of peripheral immune cell migration to the CNS after injury [17], and may contribute to BBB breakdown [48]. Treatments aimed at alleviating inflammation after TBI by inhibiting chemokine recruitment to the brain have been successful in reducing cerebral damage and cognitive deficits in animals [49,50]. Furthermore, MCP-1 and MCP-4 levels are elevated acutely after moderate and severe TBI in humans, and correlated to poor patient outcome [51,52]. While systemic chemokines have not been assessed chronically after concussion, these results are consistent with previous evidence of persistent inflammation months after mild TBI in both animals [31,53] and humans [33]. Admittedly, it is difficult to speculate whether these findings represent detrimental or reparative processes, as chemokines may also aid in neuronal repair and regenerative axonal sprouting [17,54]. Furthermore, it is unclear if MCP-1 and MCP-4 share overlapping or distinct biological actions in response to brain injury. While both molecules are involved in leukocyte recruitment, they may differ in their ability to stimulate other inflammatory mediators; for example, MCP-4 but not MCP-1 is responsible for mediating the production of chemokines IP-10 and the platelet derived chemokine ligand -5, during atherogenesis [55]. Hence, further research is needed to elucidate both the biological sequelae and health consequences of elevated systemic chemokine levels after multiple concussions in males and females.

A second important finding was tau concentrations were higher in male athletes who participate in collision sports compared to non-collision sport athletes. Additionally, when assessed in conjunction with collision sport participation, previous concussion history became a non-significant contributor to biomarker variance. This suggests that the repetitive sub-concussive impacts associated with collision sport participation may elicit a greater biological response than reported concussion, and could have a distinct pathology. Concern regarding collision sport participation and the potential link to neurodegeneration has been highlighted in recent years as tau-laden plaque depositions have been identified in the brains of post-mortem [11] and living [9] former collision-sport athletes. We found collision-sport participation in male athletes was associated with a 62% increase in peripheral tau levels compared to males who participate in non-collision sports. Previous studies have also found elevated plasma and CSF tau levels in ostensibly non-concussed male boxers [56,57] and in military personnel who sustain multiple mTBI’s during deployment [58]. Regarding the latter, tau levels were elevated in soldiers with a self-reported history of concussion, and similar to the current study, participants were sampled within a time-frame of 3 months to 3 years post-injury [58]. While it is unclear if systemic tau is pathologically related to neurodegeneration or cerebral injury, recent findings have specifically identified plasma exosomal tau as a potential CTE biomarker in former professional athletes [59], and have detected associations between plasma tau and clinical conditions such as Alzheimer’s Disease [60,61] and mTBI [62]. Taken together our results are consistent with these previous works, and suggest that systemic tau may be related to repetitive, sub-concussive impacts in male collision-sport athletes.

Brain-borne biomarkers may travel from the CNS into the periphery in at least two distinct fashions, through a disrupted/leaky blood brain barrier (BBB) [48,63], or via the glympathic system [64,65]. Regarding the latter, alterations to glymphatic function caused by clinical maladies including TBI and sleep deprivation, may attenuate the movement of proteins from the brain to the blood [55]. Yet, this process does not affect the passage of molecules across a leaky/damaged BBB [65], and while the athletes evaluated in the current study were not concussed, repetitive head impacts may alter BBB integrity and increase permeability [66,67]. Hence, it is plausible that the biomarkers we identified peripherally may be related to ongoing biological processes linked to repetitive head impacts [68,69].

An important question stemming from these findings is how the observed elevations in these indirect peripheral measures may relate to the biological consequences of repetitive head trauma, as opposed to the effects of confounding factors common to an athletic population such as exercise and/or peripheral injury. We recognize numerous inflammatory mediators, including MCP-1, may be elevated in both the plasma and skeletal muscle for hours after a single bout of exercise [70,71]. Although the time after the last exercise bout and duration/intensity were not recorded, this study was conducted during pre-season training, and we can therefore assume that all athletes (collision and non-collision) had been physical active within 72 h of blood sampling. Hence, any potential confounding effects of exercise are likely common to both groups of athletes. Furthermore, while our study design intentionally excluded athletes with musculoskeletal injuries, the physical demands of collision-sport participation may have the potential to influence biomarker concentrations. For example, tau is expressed in extracranial rat tissues [72], and in the muscle fibers of patients with inflammatory myopathy [73,74]. As tau is released from neurons as a by-product of cell death [75], muscle damage/turnover may result in the extracellular release of tau. Hence, despite being sampled before the onset of the competitive season in athletes absent overt musculoskeletal injuries, we cannot rule out the effect of pre-season training. Future studies are needed to evaluate potential extracranial release of these biomarkers, particularly from damaged/injured muscle tissue.

Though we did not identify differences in a number of previously identified TBI inflammatory markers such as IL-1β, IL-6 and IL-10, these markers have typically been evaluated in the acute stages after severe TBI [52,76–78]; conversely, our cohort was ostensibly healthy, and the median time from last concussion was approximately two years (Table 1). Furthermore, while numerous cytokines have been found elevated for up to three months after severe TBI [79], few studies have evaluated the chronic inflammatory response after concussion. Yet, Prodan and colleagues found platelet activation in previously concussed military personnel ranging from 6 months to 9 years post-injury [33], and in a follow-up study, identified a positive correlation between this inflammatory correlate and the number of concussions sustained [80]. While these previous works evaluated military personnel and included mechanistically distinct blast-related concussion, the results are consistent with our findings, and suggest that biological perturbations resulting from multiple head injuries are evident systemically up to years after injury.

In the current study, the differences identified in biomarker signatures between male and female athletes after multiple concussions is supportive of the previously noted sex-differences in immunobiology [39,40], and aligned with prior evidence of sex-differences in concussion recovery [3,35,38]. Although it is difficult to speculate on the biological basis of these findings, the potency of male and female sex hormones to differentially mediate inflammatory responses represents a plausible explanation [81]. The sexually dimorphic neurochemical composition of the brain may contribute to divergent responses to brain injury [82], leading to a different complement of proteins appearing in the blood. However, in addition to the pleiotropic effects of systemic inflammatory indices, and chemokines in particular, the gap in sex-based inflammation research in TBI makes interpretation of our results difficult. Yet, these findings necessitate sex-stratification in future concussion study cohorts, as potentially distinct mechanisms mediating the long-term effects of multiple head impacts may exist.

A limitation of the study was the cross-sectional design, therefore, we lacked the ability to evaluate inflammatory marker levels prior to injury in the athletes with a history of concussion. Furthermore, a larger sample size with additional female athletes who participate in collision sport (i.e., rugby) would allow the evaluation of biomarkers in collision vs non-collision sports. As previously identified, we were unable to control for the potential confounding effects of exercise on biomarker levels; while the homogeneity of our population suggests both collision and non-collision sport athletes were presumably similar in their exercise habits, the ability to quantify the duration and intensity of exercise and how this may have affected any of the markers assessed would have strengthened our results. Finally, while the SCAT3 is the most utilized evaluation tool in the sport context, it is a crude measure of cognitive abilities, and we recognize its comparative limitations to more advanced neuropsychological tests. However, despite these limitations, our results demonstrate potentially sex-specific systemic inflammatory alterations in athletes with multiple previous concussions, and in males who participate in collision sports.

## Conclusion

Collision sport participation in male athletes is associated with alterations in brain injury-related and inflammatory blood biomarkers. Specifically, multiple previous concussions are associated with elevations in MCP-1 in female athletes, and MCP-4 in male athletes. Furthermore, collision sport participation displays a greater covariance with systemic biomarkers compared to that of concussion history, and is specifically associated with increases in tau. Future studies are required to identify the source and biological relevance of systemic biomarkers in athletes who have sustained repetitive head trauma and who participate in collision sports, in order to better understand and characterize the potential health consequences. Particular attention should be paid to sex differences, as well as extracranial sources of biomarkers related to muscle damage and exercise.

## Supporting Information

S1 Table Biomarker values according to concussion history. (DOCX)

S2 Table Biomarker values in athletes stratified by collision sport participation. (DOCX)
