# Correction: Altered Blood Biomarker Profiles in Athletes with a History of Repetitive Head Impacts

**DOI:** 10.1371/journal.pone.0164912

**Published:** 2016-10-11

**Authors:** Alex P. Di Battista, Shawn G. Rhind, Doug Richards, Nathan Churchill, Andrew J. Baker, Michael G. Hutchison

The penultimate sentence of the Measures section should have cited reference 41 instead of 39.

In the Funding section, the name of the funder CIMVHR should be listed as “Canadian Institute for Military and Veteran Health Research (CIMVHR)”.
