# Supplementary material for: Altered Blood Biomarker Profiles in Athletes with a History of Repetitive Head Impacts
Source: PLoS One. 2016 Jul 26;11(7):e0159929. doi: 10.1371/journal.pone.0159929 (PMC4961456; doi:10.1371/journal.pone.0159929)
Supplement: S1 Table — (DOCX) [file pone.0159929.s001.docx]

**S1 Table.** Biomarker values according to concussion history.

| **Markers (pg/mL)** | **Males (n = 60)** | | **Females (n = 27)** | |
| --- | --- | --- | --- | --- |
|  | **No concussion history**  **(n = 38)** | **Concussion history**  **(n = 22)** | **No concussion history**  **(n = 15)** | **Concussion history**  **(n = 12)** |
| *Cytokines* | | | | |
| IL-1α | -- | -- | -- | -- |
| IL-1β | -- | -- | -- | -- |
| IL-2 | -- | -- | -- | -- |
| IL-4 | -- | -- | -- | -- |
| IL-5 | -- | -- | -- | -- |
| IL-6 | -- | -- | -- | -- |
| IL-7 | 2.1 (1.8 – 3.5) | 3.7 (2.9 – 5.0) | 2.2 (2.0 – 2.9) | 2.9 (2.4 – 3.6) |
| IL-10 | -- | -- | -- | -- |
| IL-12p40 | 120.6 (93.7 – 140.9) | 113.5 (77.9 – 142.5) | 132.2 (104.1 – 154.0) | 136.8 (80.6 – 163.8) |
| IL-12p70 | -- | -- | -- | -- |
| IL-13 | -- | -- | -- | -- |
| IL-15 | 2.3 (2.0 – 2.8) | 2.2 (2.0 – 2.7) | 2.4 (1.9 – 2.7) | 2.4 (2.2 – 2.6) |
| IL-16 | 304.9 (191.4 – 374.6) | 305.9 (224.9 – 395.2) | 205.9 (186.0 – 220.3) | 237.8 (167.8 – 318.1) |
| IL-17A | -- | -- | -- | -- |
| TNF-α | 1.8 (1.5 – 2.2) | 1.9 (1.7 – 2.1) | 1.8 (1.5 – 2.3) | 1.7 (1.5 – 2.2) |
| TNF-β | -- | -- | -- | -- |
| GM-CSF | -- | -- | -- | -- |
| VEGF | 34.6 (27.5 – 51.1) | 37.4 (29.8 – 55.6) | 35.6 (26.7 – 65.0) | 39.1 (26.7 – 56.6) |
| IFN-γ | -- | -- | -- | -- |
| *Chemokines* | | | | |
| Eotaxin | 78.1 (60.2 – 101.2) | 83.6 (68.5 – 101.7) | 77.7 (62.2 – 87.8) | 69.8 (43.0 – 81.6) |
| Eotaxin-3 | 21.7 (18.5 – 28.1) | 23.4 (21.5 – 43.3) | -- | 18.9 (14.5 – 36.6) |
| IP-10 | 207.1 (162.9 – 247.6) | 207.0 (156.4 – 264.2) | 200.0 (148.3 – 258.4) | 190.2 (182.1 – 297.7) |
| IL-8 | 1.7 (1.5 – 2.6) | 2.5 (1.6 – 4.1) | 2.3 (1.7 – 2.6) | 1.6 (1.3 – 2.9) |
| MCP-1 | 89.9 (76.5 – 117.4) | 98.6 (84.7 – 116.9) | 69.3 (58.4 – 90.3) | 85.5 (71.1 – 102.9) |
| MCP-4 | 26.5 (18.7 – 34.0) | 31.9 (23.9 – 54.7) | 20.2 (16.8 – 24.7) | 26.1 (19.0 – 38.4) |
| MDC | 809.4 (706.1 – 924.3) | 824.9 (711.9 – 1063.2) | 828.0 (676.6 – 1073.7) | 760.5 (710.4 – 1003.9) |
| MIP-1α | -- | -- | -- | -- |
| MIP-1β | 37.8 (32.7 – 49.4) | 42.4 (32.9 – 54.5) | 31.4 (29.2 – 41.9) | 42.6 (26.5 – 52.1) |
| TARC | 39.2 (29.2 – 68.4) | 43.2 (23.8 – 58.5) | 36.1 (25.2 – 53.5) | 49.4 (24.4 – 55.5) |
| *Brain injury* | | | | |
| s100B | 698.4 (591.2 – 858.0) | 738.8 (669.4 – 946.9) | 659.3 (603.0 – 754.7) | 640.4 (516.1 – 823.8) |
| GFAP | 75.9 (63.9 – 83.2) | 69.9 (64.4 – 91.1) | 102.7 (98.1 – 124.2) | 62.0 (56.6 – 78.1) |
| NSE (ng/mL) | 1.6 (1.2 – 2.1) | 1.7 (1.4 – 2.4) | 1.5 (1.1 – 2.1) | 1.0 (1.0 – 1.4) |
| Neurogranin (ng/mL) | 7.3 (4.5 – 11.8) | 8.0 (6.1 – 11.0) | 6.7 (2.6 – 13.6) | 9.9 (4.6 – 11.4) |
| CKBB | -- | -- | -- | -- |
| VILIP-1 | -- | -- | -- | -- |
| Tau | 25.9 (21.5 – 45.5) | 25.6 (20.7 – 33.9) | 22.2 (12.3 – 25.6) | 20.8 (14.7 – 27.3) |
| vWF (μg/ml) | 34.6 (24.1 – 43.8) | 41.7 (18.3 – 54.9) | 44.3 (24.8 – 57.5) | 42.5 (24.8 – 58.5) |
| BDNF | 851.6 (507.5 – 1910.7) | 902.1 (570.9 – 1758.8) | 766.1 (390.5 – 2031.8) | 945.1 (730.6 – 2445.2) |
| PRDX-6 (ng/mL) | 27.5 (18.2 – 34.0) | 29.0 (26.3 – 39.2) | 23.7 (17.8 – 27.1) | 20.0 (16.0 – 24.2) |

Interleukin (IL) -1α, -1β, -2, -4, -5, -6, -7, -10, -12p40, -12p70, -13, -15, -16, -17A, tumor necrosis factor (TNF) -α, -β, granulocyte macrophage colony-stimulating factor (GM-CSF), vascular endothelial growth factor (VEGF), interferon-gamma (IFN-γ), eotaxin, eotaxin-3, interferon gamma-induced protein (IP) -10, IL-8. monocyte chemoattractant protein (MCP) -1, -4, macrophage derived chemokine, (MDC),

thymocyte- and activation-regulated chemokine (TARC), s100 calcium binding protein beta (s100B), glial fibrillary acidic protein (GFAP), neuron specific enolase (NSE), creatine kinase-BB isoenzyme (CKBB), visinin-like protein (VILIP-1), von Willebran factor (vWF), brain derived neurotrophic factor (BDNF), peroxiredoxin (PRDX) -6.

^*^ = all markers reported as pg/mL unless otherwise stated

“--” = below assay quantitation in >50% of samples analyzed.
