# Supplementary material for: Altered Blood Biomarker Profiles in Athletes with a History of Repetitive Head Impacts
Source: PLoS One. 2016 Jul 26;11(7):e0159929. doi: 10.1371/journal.pone.0159929 (PMC4961456; doi:10.1371/journal.pone.0159929)
Supplement: S2 Table — (DOCX) [file pone.0159929.s002.docx]

| **Markers (pg/mL)*** | **Non-collision sport (n = 46)** | | **Collision sport (n = 41)** |
| --- | --- | --- | --- |
|  | **Female (n = 25)** | **Male (n = 21)** | **Male (n = 39)** |
| *Cytokines* | | | |
| IL-1α | -- | -- | -- |
| IL-1β | -- | -- | -- |
| IL-2 | -- | -- | -- |
| IL-4 | -- | -- | -- |
| IL-5 | -- | -- | -- |
| IL-6 | -- | -- | -- |
| IL-7 | 2.4 (2.1 – 3.6) | 2.7 (1.9 – 4.5) | 2.7 (1.8 – 4.4) |
| IL-10 | -- | -- | -- |
| IL-12p40 | 132.2 (104.1 – 160.2) | 124.0 (102.0 – 138.4) | 113.5 (77.8 – 142.1) |
| IL-12p70 | -- | -- | -- |
| IL-13 | -- | -- | -- |
| IL-15 | 2.5 (2.1 – 2.7) | 2.2 (2.0 – 2.6) | 2.2 (2.0 – 2.7) |
| IL-16 | 214.5 (176.9 – 290.0) | 250.6 (207.2 – 349.7) | -- |
| IL-17A | -- | -- | -- |
| TNF-α | 1.8 (1.5 – 2.2) | 1.9 (1.4 – 2.4) | 1.9 (1.7 – 2.1) |
| TNF-β | -- | -- | -- |
| GM-CSF | -- | -- | -- |
| VEGF | 36.5 (23.5 – 48.5) | 38.7 (30.8 – 59.9) | 34.1 (26.6 – 55.5) |
| IFN-γ | -- | -- | -- |
| *Chemokines* | | | |
| Eotaxin | 73.6 (62.2 – 86.2) | 83.9 (64.0 – 101.2) | 77.5 (63.3 – 103.3) |
| Eotaxin-3 | -- | 22.2 (20.4 – 32.0) | 23.1 (19.6 – 31.3) |
| IP-10 | 183.4 (148.4 – 297.7) | 218.4 (189.1 – 277.2) | 202.6 (155.6 – 248.0) |
| IL-8 | 2.1 (1.5 – 2.6) | 2.0 (1.5 – 2.7) | 1.8 (1.5 – 2.8) |
| MCP-1 | 79.3 (65.1 – 95.4) | 84.1 (65.3 – 92.8) | 102.3 (84.7 – 135.3) |
| MCP-4 | 23.0 (16.8 – 38.3) | 28.1 (24.1 – 41.1) | 26.5 (18.4 – 41.2) |
| MDC | 773.1 (701.5 – 1043.6) | 790.8 (658.3 – 930.0) | 825.0 (713.4 – 989.1) |
| MIP-1α | -- | -- | -- |
| MIP-1β | 39.6 (27.4 – 48.9) | 43.1 (32.7 – 54.2) | 37.8 (33.5 – 49.3) |
| TARC | 42.2 (25.2 – 54.5) | 42.8 (27.3 – 88.2) | 42.2 (29.4 – 53.5) |
| *Brain injury* | | | |
| s100B | 651.6 (578.9 – 764.6) | 654.1 (585.8 – 746.6) | 804.1 (642.7 – 929.3) |
| GFAP | 87.0 (62.3 – 110.9) | 68.2 (58.1 – 99.4) | 76.4 (64.9 – 82.5) |
| NSE (ng/mL) | 1.3 (1.0 – 1.6) | 1.6 (1.2 – 2.1) | 1.6 (1.3 – 2.1) |
| Neurogranin (ng/mL) | 8.1 (3.5 – 12.0) | 7.0 (3.6 – 12.5) | 8.0 (5.3 – 10.8) |
| CKBB | -- | -- | -- |
| VILIP-1 | -- | -- | -- |
| Tau | 20.1 (13.6 – 26.9) | 21.0 (15.5 – 22.3) | 33.9 (24.4. – 50.1) |
| vWF (μg/mL) | 44.3 (24.8 – 63.6) | 24.6 (17.3 – 37.7) | 42.5 (24.3 – 57.5) |
| BDNF | 969.4 (631.2 – 2031.8) | 1219.2 (688.7 – 2480.1) | 734.0 (519.1 – 1535.1) |
| PRDX-6 (ng/mL) | 44.3 (24.8 – 63.6) | 27.5 (18.6 – 33.3) | 29.5 (20.8 – 35.9) |

**S2 Table.** Biomarker values in athletes stratified by collision sport participation.

Interleukin (IL) -1α, -1β, -2, -4, -5, -6, -7, -10, -12p40, -12p70, -13, -15, -16, -17A, tumor necrosis factor (TNF) -α, -β, granulocyte macrophage colony-stimulating factor (GM-CSF), vascular endothelial growth factor (VEGF), interferon-gamma (IFN-γ), eotaxin, eotaxin-3, interferon gamma-induced protein (IP) -10, IL-8. monocyte chemoattractant protein (MCP) -1, -4, macrophage derived chemokine, (MDC),

thymocyte- and activation-regulated chemokine (TARC), s100 calcium binding protein beta (s100B), glial fibrillary acidic protein (GFAP), neuron specific enolase (NSE), creatine kinase-BB isoenzyme (CKBB), visinin-like protein (VILIP-1), von Willebran factor (vWF), brain derived neurotrophic factor (BDNF), peroxiredoxin (PRDX) -6.

^*^ = all markers reported as pg/mL unless otherwise stated

“--” = below assay quantitation in >50% of samples analyzed.
